# Comparison of classical method and microdebrider technique for adenoidectomy in pediatric patients with obstructive sleep apnea

**DOI:** 10.20407/fmj.2023-009

**Published:** 2024-02-15

**Authors:** Hiroya Inada, Masatoshi Hirata, Ayami Kimura, Satoshi Ito, Kazuki Shikano, Masamichi Kaneko, Takayuki Okano, Seiichi Nakata

**Affiliations:** 1 Department of Otorhinolaryngology and Sleep Medicine, Fujita Health University Bantane Hospital, Nagoya, Aichi, Japan; 2 Department of Clinical Laboratory, Fujita Health University Bantane Hospital, Nagoya, Aichi, Japan

**Keywords:** Obstructive sleep apnea, Microdebrider, Adenoidectomy, AHI

## Abstract

**Objectives:**

The aim of this study was to evaluate the safety and efficacy of microdebrider adenoidectomy on sleep-disordered breathing among pediatric patients with OSA.

**Methods:**

In the microdebrider group (Group I), there were 30 Japanese OSA patients consisting of 26 boys and 4 girls. For comparison, we had 15 children (13 boys and 2 girls) who underwent classical adenoidectomy (Group II). Patients in Group I were selected from a pool of 95 pediatric Japanese OSA patients and were matched by age, preoperative AHI, and Kaup index with those in Group II.

Parameters such as the amount of residual adenoid tissue, bleeding, duration of the procedure, and sleep-related metrics were compared between the two groups.

**Results:**

A significant improvement in postoperative AHI was observed in Group I (*p*<0.05). The prevalence of AHI <1 was significantly higher in Group I compared with Group II (*p*<0.05). Additionally, the amount of postoperative residual adenoid was significantly less in Group I (3/30 of Grade 3 and 4 adenoid size) than in Group II (7/15, *p*<0.05). Furthermore, a reduction in postoperative AHI was proportionally associated with a decrease in residual adenoid.

**Conclusions:**

The newly developed microdebrider adenoidectomy technique for pediatric OSA patients with adenotonsillar hypertrophy demonstrated greater accuracy and efficacy in ameliorating sleep apnea symptoms compared with the standard adenoidectomy approach.

## Introduction

Adenoid hypertrophy is a common cause of nasal obstruction in the pediatric population, often leading to recurrent otitis media or Eustachian tube dysfunction. Recent evidence also suggests that this condition can precipitate obstructive sleep apnea (OSA). The standard treatment for this condition in the pediatric population is adenoidectomy, either as a standalone procedure or in conjunction with tonsillectomy.^1^ Indications for this surgery include adenotonsillar hypertrophy, nasal obstruction, recurrent effusive otitis media, and obstructive sleep apnea. Historically, the procedure has evolved from rudimentary techniques involving fingernails, steel nails, and cutting or biting forceps, to the use of specialized instruments like adenotomes and adenoid curettes.^2^ The standard direct adenoidectomy, first established by Guggenheim in 1957 and later modified by Beckman and La Force,^3^ has been widely utilized until recently.

However, these conventional adenoidectomy techniques are performed blindly, not under direct visual guidance, leading to potential issues such as postoperative residual adenoid tissue. This limitation is a significant drawback of these traditional methodologies. Moreover, perioperative bleeding remains a risk, one that even skilled otorhinolaryngologists cannot always prevent.^4^

Therefore, the need for a more sophisticated and safer approach to adenoidectomy, performed under direct vision, has been increasingly recognized among otorhinolaryngologists. To address this need, a variety of instruments have been implemented, including the electronic molecular resonance tool, suction diathermy, microdebrider, and laser.^5–7^ Recent advancements have led to the development of microdebrider adenoidectomy, which utilizes a laryngeal auto-fiberscope to visualize the entire oro/naso-pharynx. Studies have shown that this technique is effective in reducing both postoperative residual adenoids and perioperative bleeding compared with conventional adenoidectomy.^8,9^ Therefore, the microdebrider technique holds the potential to revolutionize adenoidectomy procedures for otorhinolaryngologists.^10,11^

Anatomical constriction of the upper airway, often caused by hypertrophied adenoids and tonsils, can be a precipitating factor for OSA. Surgical removal of these redundant tissues has been known to reduce the occurrence of sleep apnea events in pediatric populations. In light of this, this study aims to evaluate the safety and efficacy of microdebrider adenoidectomy in treating sleep-disordered breathing among pediatric patients with OSA.

## Methods

### Patients

We conducted a retrospective review of medical records for children who had undergone adenoidectomy or adenotonsillectomy for the treatment of OSA. This study was classified as exempt by the local institutional review board, as it collected data during the course of standard treatment for OSA. Group I, the microdebrider group, consisted of 30 Japanese children (26 boys and 4 girls), with an average age of 4.07±1.26 years (mean±SD). These children were referred to Fujita Health University 2nd Hospital between 2015 and 2017 and were diagnosed with either adenotonsil hypertrophy or adenoid vegetation. Accordingly, adenotonsillectomy or adenoidectomy was indicated and performed using the microdebrider technique by experienced otorhinolaryngologists. For comparative analysis, Group II included 15 Japanese children treated with the classical adenoidectomy technique. Group I participants were selected from a pool of 95 pediatric OSA patients whose medical records, including pre- and postoperative polysomnography, were available for the three-year period from January 2015 to December 2017. The 30 selected children were age-matched and had similar preoperative apnea-hypopnea index (AHI) and Kaup index values to those in Group II. Statistical analyses were conducted to investigate correlations between otorhinolaryngologic findings, age, obesity index, and pre- and postoperative sleep parameters such as AHI, lowest oxygen saturation (L-SpO_2_) and 3% oxygen desaturation index (3% ODI). Morphological findings included grading of tonsil sizes based on a set scale (0, tonsil in the fossa; 1, tonsil occupies less than 25% of the oropharynx; 2, tonsil occupies between 25% to 50% of the oropharynx; 3, tonsil occupies between 50% to 75% of the oropharynx; 4, tonsil occupies more than 75% of the oropharynx).^12^ Similarly, adenoid size was graded according to nasal endoscopy findings (1, adenoid tissue not in contact with adjacent structures; 2, adenoid tissue in contact with torus tubaris; 3, adenoid tissue in contact with vomer; 4, adenoid tissue in contact with the soft palate).^13^ These gradings were determined by a designated otorhinolaryngologist who was blinded to the patient’s information.

Concerning the grading of adenoid tissue, we utilized a fiberscope to capture detailed video or descriptive records of the epipharynx, including the adenoid. This enabled us to employ the new grading system proposed by Sanjay et al.^13^

No significant preoperative differences were observed between the two groups in terms of age, sex, or Kaup index, as shown in Table 1. For Group I, 30 adenotonsillectomies were performed, while 15 were performed for Group II. All participants underwent postoperative follow-up at six-month intervals for at least one year. During these postoperative inspections, no residual tonsil tissue was identified in either Group I or Group II.

### Surgical method

**Figure 1:**
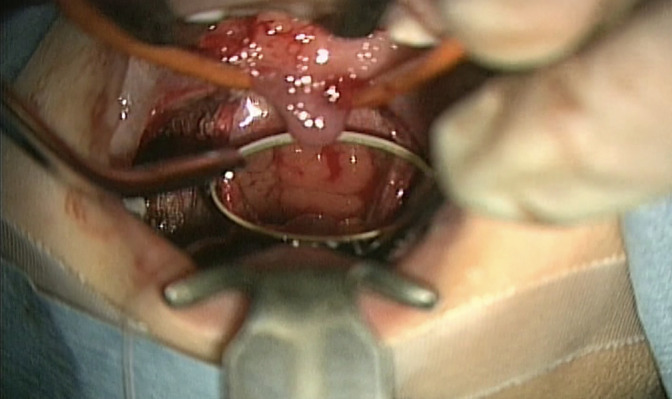
Initial elevation of the soft palate using a flexible catheter to facilitate the insertion of the laryngeal mirror from the oral cavity into the pharynx.

**Figure 2:**
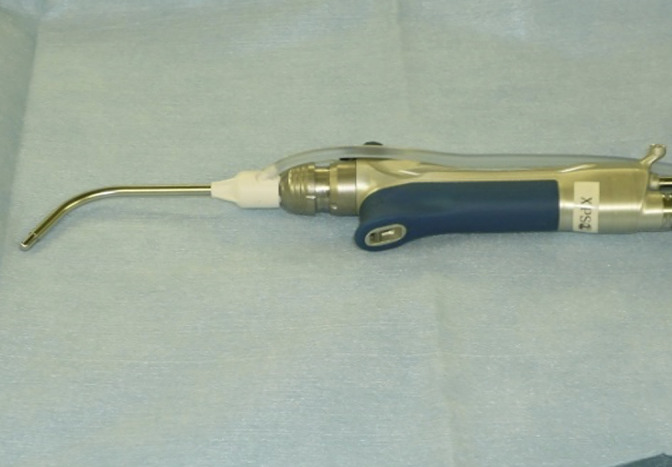
Microdebrider and curved shaver blade.

Microdebrider adenoidectomy was performed under general anesthesia. First, the soft palate was elevated using a flexible catheter to facilitate the insertion of a laryngeal mirror from the oral cavity to the pharynx. With the adenoid visualized under the laryngeal mirror, Xylocaine^®^ containing 1% epinephrine was directly injected for local anesthesia. Finally, tissue was resected and aspirated using a microdebrider (Figures 1 and 2).

Two specific otorhinolaryngologists performed the microdebrider adenoidectomy procedures for Group I patients. For Group II patients, these two otorhinolaryngologists were joined by three additional operators to perform the classical adenoidectomy. The inter-operator variations in the procedures and outcomes were minimal, owing to the well-established techniques. In addition, a designated supervisor reviewed and, if necessary, corrected the procedures.

### Sleep study

The study employed standard overnight polysomnography without the use of sedatives or sleep deprivation, in accordance with guidelines of the American Thoracic Society.^14^ Polysomnography was conducted using ALICE3 (Respironics Inc., Murrysville, Pennsylvania, United States) and included pulse oximetry for all subjects. Electroencephalogram (EEG) channels C3-A2, C4-A1, O1-A2, and O2-A1, along with electrooculogram, electromyograms (EMG) from the mentalis, legs, and diaphragm, and electrocardiograms in bipolar CM5 and standard V5 lead positions, were recorded. Respiration was monitored via an oronasal thermistor and a thoracoabdominal piezo sensor. All recordings and sleep stage scorings were independently verified by designated polysomnographers who were blinded to the patient’s information. Sleep stages were manually scored based on the Rechtshaffen and Kales criteria,^15^ and the contributions of stages 1, 2, 3+4, and rapid eye movement (REM) sleep to the total sleep time were calculated. An apnea was defined as a cessation of airflow for longer than two respiratory cycles despite breathing effort. Hypopnea was defined as a 50% to 80% reduction of airflow accompanied by either a 3% or greater desaturation or an arousal. Metrics such as AHI, L-SpO_2_, 3% ODI, and number of arousals per hour (arousal index) were calculated. Although an AHI of 5/h or higher is generally indicative of OSA in adults,^16^ an apnea index greater than 1 was considered statistically abnormal for pediatric OSA in this study. ^17^

### Statistical analysis

Data are presented as means±standard deviation (SD). A probability (*p*) value less than 0.05 was considered statistically significant. Statistical analysis was performed using SPSS 15.0 (SPSS Inc.; Chicago, IL). The χ^2^ test, with Yates’ and William’s corrections, was employed for 2×2 and 2×4 tables to compare the distribution of the stage of residual adenoid.

## Results

No difference was observed in the duration of the procedure or the amount of bleeding between Group I and Group II (62.6±5.6 min vs. 61.1±5.8, 15.1±3.9 vs. 10.7±3.8 mL). This is consistent with previous studies that have indicated microdebrider adenoidectomy to be either equivalent or superior to classical adenoidectomy in these aspects.

**Figure 3:**
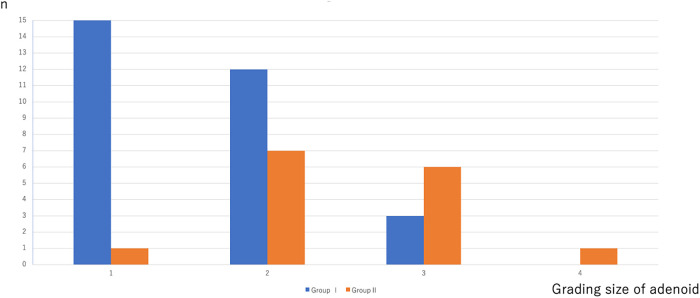
Distribution of individuals post-surgery based on adenoid size grading in groups I and II (number of cases/total number in group).

**Figure 4:**
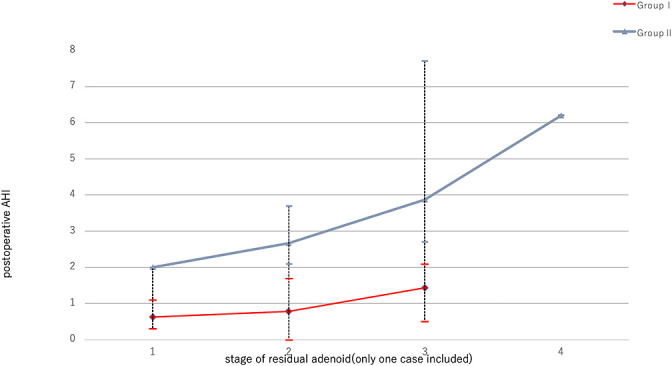
Mean postoperative AHI in groups I and II according to stage of residual adenoid tissue.

Conversely, a significant difference was noted between Group I and Group II when adenoid sizes of grades 3 and 4 were assessed (3/30 vs. 7/15, *p*<0.05) (Figure 3). The postoperative AHI was lower in Group I compared with Group II, correlating with less residual adenoid tissue (Figure 4).

As shown in Table 2, a significant improvement in postoperative AHI was observed in Group I compared with Group II (*p*<0.05). Interestingly, the prevalence of cases with an AHI of less than 1 was significantly higher in Group I than in Group II (*p*<0.05). However, there was no difference in the degree of improvement in L-SpO_2_ between the two groups.

## Discussion

Compared with the conventional curettage method, the microdebridder technique has been shown to result in less residual adenoid tissue and more favorable impact on the occurrence of OSA in pediatric patients with adenotonsillar hypertrophy.

Figure 4 illustrates that the mean AHI following microdebrider adenoidectomy was lower than that observed after the classical operation. This reduction correlated with the amount of residual adenoid tissue. These findings suggest that microdebrider adenoidectomy is more effective in ameliorating sleep apnea symptoms than the classical method, even when the amount of residual adenoid tissue is equivalent. Furthermore, the results imply that the microdebrider technique is superior to the classical method, as it allows for more efficient resection of the critical adenoid anatomy implicated in the genesis of OSA, such as the posterior nasal aperture, under direct vision.

It is worth noting that microdebrider adenoidectomy was able to effectively eliminate sleep apneas, achieving a minimal postoperative AHI of less than 1. This is particularly significant given that an AHI of 1 or greater is generally considered indicative of OSA in the pediatric population.^18,19^

In terms of alternative surgical techniques for adenoidectomy, we opted not to employ suction diathermy in favor of electrocautery adenoidectomy (ECA). This decision was based on reports indicating that postoperative regrowth of adenoid tissue is more frequently observed following the use of suction diathermy compared with the conventional curettage method.^20^

One limitation of our study may be the conflation of adenoidectomy and adenotonsillectomy cases in both Groups I and II. However, postoperative evaluations showed no residual palatine tonsil in either group, which allows us to mitigate the effects of the two different surgical procedures on the outcomes. In this context, we believe that residual adenoid tissue significantly contributes to postoperative sleep apneas occurrences in our pediatric OSA patients.

From an economic perspective, the microdebrider method for adenoidectomy is currently more costly than traditional techniques. For example, the powered RADenoid^®^ blade (18-84008, Medtronic Xomed, Inc., Jacksonville, Florida) is approximately $97, while the hand-switching ValleyLab^®^ suction coagulator is priced at around $11.

In conclusion, the newly developed microdebrider method for adenoidectomy in pediatric patients with OSA and adenotonsillar hypertrophy demonstrates greater accuracy and efficacy in ameliorating sleep apnea compared with conventional adenoidectomy techniques.

## Figures and Tables

**Table1:** Demographic Characteristics

Surgical method	Microdebridder (n=30)	Curette (n=15)	
Median age, yrs	4.07±1.26	3.93±1.24	N.S.
Sex: M/F	26/4	13/2	N.S.
AHI	17.09±14.96	17.51±13.76	N.S.
Kaup index	15.54±1.45	15.79±1.05	N.S.

**Table2:** Postoperative AHI (Group 1 vs Group 2)

	Group 1			Group 2	
	pre-Ope	post-Ope		pre-Ope	post-Ope
AHI (/h)	17.09±14.96	0.77±0.49*		17.51±13.76	3.33±1.54*
L-SpO_2_ (%)	88.1±4.9	93.47±2.77		83.1±8.7	90.27±4.11
The ratio of AHI <1		23/30*			0/15*

* p<0.05
